# Similar Neural Activity during Fear and Disgust in the Rat Basolateral Amygdala

**DOI:** 10.1371/journal.pone.0027797

**Published:** 2011-12-14

**Authors:** Steven J. Shabel, Will Schairer, Rachel J. Donahue, Victoria Powell, Patricia H. Janak

**Affiliations:** 1 Ernest Gallo Clinic and Research Center, University of California San Francisco, Emeryville, California, United States of America; 2 Neuroscience Graduate Program, University of California San Francisco, San Francisco, California, United States of America; 3 Wheeler Center for the Neurobiology of Addiction, University of California San Francisco, San Francisco, California, United States of America; 4 Department of Neurology, University of California San Francisco, San Francisco, California, United States of America; University of Leuven, Belgium

## Abstract

Much research has focused on how the amygdala processes individual affects, yet little is known about how multiple types of positive and negative affects are encoded relative to one another at the single-cell level. In particular, it is unclear whether different negative affects, such as fear and disgust, are encoded more similarly than negative and positive affects, such as fear and pleasure. Here we test the hypothesis that the basolateral nucleus of the amygdala (BLA), a region known to be important for learned fear and other affects, encodes affective valence by comparing neuronal activity in the BLA during a conditioned fear stimulus (fear CS) with activity during intraoral delivery of an aversive fluid that induces a disgust response and a rewarding fluid that induces a hedonic response. Consistent with the hypothesis, neuronal activity during the fear CS and aversive fluid infusion, but not during the fear CS and rewarding fluid infusion, was more similar than expected by chance. We also found that the greater similarity in activity during the fear- and disgust-eliciting stimuli was specific to a subpopulation of cells and a limited window of time. Our results suggest that a subpopulation of BLA neurons encodes affective valence during learned fear, and furthermore, within this subpopulation, different negative affects are encoded more similarly than negative and positive affects in a time-specific manner.

## Introduction

The basolateral nucleus of the amygdala (BLA) is important for affective processing [1]–[5] and has a well-established role in the acquisition and expression of Pavlovian fear memories [6]. Although fear conditioning changes the responses of many neurons in the BLA to a conditioned fear stimulus (CS) [7], consistent with affective valence encoding [8]–[10], these neuronal responses could encode other attributes of the stimulus, including the sensory properties of the outcome it predicts [11], [12], its arousing or activating properties [9], [10], [13]–[15], and/or the discrete affect induced by the stimulus (i.e., fear). Similarly, although previous studies found changes in neuronal activity in the amygdala when the value of stimuli was changed from positive to negative (or vice versa) [8], [12], consistent with valence encoding, these changes could also reflect changes in the sensory properties of the outcome, changes in arousal, and/or changes in the discrete affect induced by the stimulus (e.g., disgust to desire). Thus, it is possible that similar changes in activity would have occurred if the stimuli were manipulated to induce a different affect of the same affective valence (e.g., disgust to fear).

**Figure 1:**
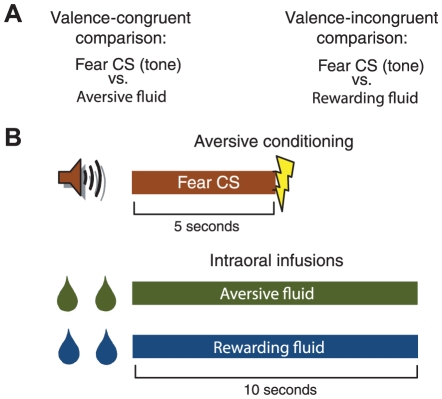
Experimental design and methods. (**A**) Experimental design. If the BLA encodes emotional valence during learned fear, then neuronal activity should be more similar during the fear CS and aversive fluid infusion (valence-congruent comparison) than during the fear CS and rewarding fluid infusion (valence-incongruent comparison). (**B**) Methods. Neuronal activity during the 5 second fear CS was compared to neuronal activity during the first 10 seconds of the rewarding and aversive intraoral fluid infusions.

Here, we test the hypothesis that the BLA encodes affective valence during learned fear by comparing neuronal activity during a fear CS with neuronal activity during an aversive fluid infusion that elicits a disgust response (valence-congruent comparison) and neuronal activity during a rewarding fluid infusion that elicits a hedonic response (valence-incongruent comparison; Fig. 1A). If the BLA encodes affective valence during learned fear, then neuronal activity during the fear CS should be more similar to neuronal activity during the aversive fluid infusion than the rewarding fluid infusion. Note that because both the valence-congruent and valence-incongruent comparisons compare two different affects, any differences in the comparisons cannot be attributed to a simple change in affect.

## Results

### Behavior and blood pressure responses to affective stimuli

We implanted rats (n = 7) with electrode arrays for recording single-cell activity in the BLA, a telemetric, abdominal transmitter for monitoring blood pressure and movement, and three intraoral cannulae for infusion of an aversive fluid, a rewarding fluid, and water rinses between each aversive and rewarding fluid infusion. Test sessions consisted of three parts – 20 fear conditioning trials (each trial consisted of a 5 second tone followed by a brief, mild electric footshock), 20 rewarding fluid infusions, and 20 aversive fluid infusions (Fig. 1B and Fig. S1) – and were conducted while rats were water restricted. The aversive fluid was either a salt (n = 4 rats) or sucrose solution (n = 3) that was made aversive via previous pairing with injections of lithium chloride, a nausea/malaise-inducing drug that is commonly used for conditioning taste aversions. This procedure elicits rejection/disgust responses during subsequent fluid infusions [16], [17] and engages the BLA [18]–[22], although the precise role of the BLA in conditioned taste aversion remains unclear [23]. The rewarding fluid was conversely either a sucrose (n = 4 rats) or salt (n = 3) solution that was previously paired with benign injections of saline as a control for the lithium chloride conditioning injections. Note that since the rats were water restricted, they readily consumed both the sucrose and salt solutions.

**Figure 2:**
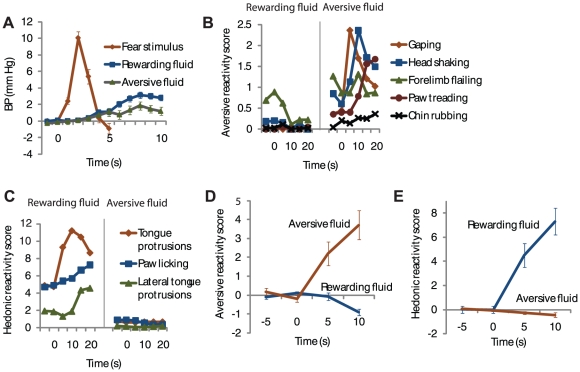
Blood pressure and behavior during the recording sessions. (**A**) Change in blood pressure during fear CS, rewarding fluid infusion, and aversive fluid infusion (n = 39 sessions). (**B**) Mean of individual types of aversive taste reactivity responses during rewarding and aversive fluid infusions. (n = 45 sessions). (**C**) Mean of individual types of positive hedonic taste reactivity responses during rewarding and aversive fluid infusions. (n = 45 sessions). (**D**) Change in aversive taste reactivity responses shown in (**B**) (n = 45 sessions). (**E**) Change in positive hedonic taste reactivity responses shown in (**C**) (n = 45 sessions). Values represent mean +/− standard error of the mean.

As shown previously [14], [24]–[26], pairing a CS with footshock increased blood pressure during the CS compared to habituation trials that were given during one session before fear conditioning began (habituation, ΔBP, −.3±.4 mm Hg; conditioning, ΔBP, 3.944±1.1 mm Hg; n = 6 rats; paired *t* test; *P* = .028)(Fig. 2A). After conditioning, rats showed species-specific fear responses during the CS [27] – escape/fleeing responses during the conditioning sessions (pre-CS, .05±.02 movement counts; CS, .35±.11; n = 6 rats; paired *t* test; *P* = .015)(Fig. S2) and freezing during a single test session without footshock given at the end of the experiment (pre-CS, 1.4±1.5% freezing; CS, 71.4±5.0% freezing; n = 7 rats; paired *t* test; *P* = 7.2×10^−6^).

Infusion of the aversive and rewarding fluids also elicited increases in blood pressure during the recording sessions, though the increases were not as large as during the fear CS (rewarding fluid, ΔBP, 1.7±.3 mm Hg; aversive fluid, ΔBP, .9±.3 mm Hg; fear CS, ΔBP, 3.5±.5 mm Hg; paired *t* tests, n = 39 sessions: rewarding fluid vs. fear CS, *P* = .004; aversive fluid vs. fear CS, *P* = 4.5×10^−5^; rewarding fluid vs. aversive fluid, *P* = .07)(Fig. 2A). The rats clearly discriminated between the aversive fluid and rewarding fluid during the recording sessions, as indicated by differences in (aversive) rejection and (hedonic) ingestion taste reactivity responses that have been extensively characterized elsewhere [28], [29] (Fig. 2B,C). As expected, the aversive fluid elicited an increase in aversive taste reactivity responses (paired *t* test, n = 45 recording sessions, *P* = 2.5×10^−5^)(Fig. 2D) and a decrease in positive hedonic taste reactivity responses (paired *t* test, *P* = .005)(Fig. 2E), while the rewarding fluid elicited an increase in positive hedonic taste reactivity responses (paired *t* test, *P* = 4.4×10^−7^)(Fig. 2E) and a decrease in aversive reactivity responses (paired *t* test, *P* = .004) (Fig. 2D). Thus, the fear CS, the aversive fluid, and the rewarding fluid elicited three different affective responses which likely correspond to human forms of fear, disgust, and pleasure, respectively.

### Subpopulation-specific valence encoding

**Figure 3:**
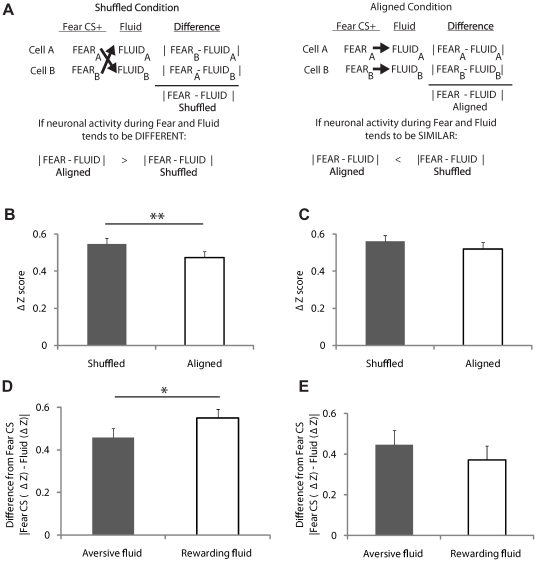
Neuronal activity during affective stimuli. (**A**) Design of aligned versus shuffled comparisons. If neuronal activity is similar during the affective stimuli, then the difference in the aligned condition should be less than the difference in the shuffled condition. If neuronal activity is different during the affective stimuli, then the difference in the aligned condition should be greater than the difference in the shuffled condition. (**B**) Valence-congruent comparison. Activity during fear CS and aversive fluid infusion is more similar than expected by chance (n = 84 cells). (**C**) Valence-incongruent comparison. Activity during fear CS and rewarding fluid infusion is not more similar than expected by chance (n = 84 cells). (**D**) Non-SD cells. Valence-congruent comparison of activity is more similar than valence-incongruent comparison (n = 62 cells). (**E**) SD cells. No significant difference between valence-congruent and valence-incongruent comparison of activity (n = 22 cells). Values represent mean +/− standard error of the mean. * *P*<.01, ** *P*<.0001.

**Figure 4:**
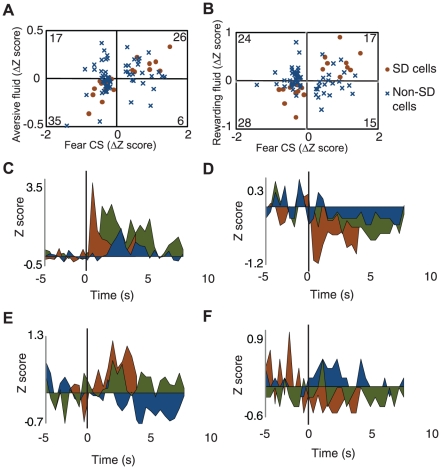
Population plots and examples of SD and non-SD cells. (**A**) Population plot of changes in neuronal activity during the fear CS and aversive fluid infusion. (**B**) Population plot of changes in neuronal activity during the fear CS and rewarding fluid infusion. (**C**)–(**F**) depict average activity to each of the stimuli for one cell. Red is fear CS, blue is rewarding fluid infusion, and green is aversive fluid infusion. (**C**) Example perievent time histogram of an SD cell which had an increase in activity during the fear CS. Mean and standard deviation of firing rates for baseline periods before fear CS, rewarding fluid infusion, and aversive fluid infusion, respectively: 2.0±2.3; 2.0±2.1; 1.6±1.7; (**D**) Example perievent time histogram of an SD cell which had a decrease in activity during the fear CS. Mean and standard deviation of firing rates for baseline periods before fear CS, rewarding fluid infusion, and aversive fluid infusion, respectively: 2.1±1.5; 0.3±0.7; 0.5±0.6; (**E**) Example perievent time histogram of a non-SD cell which had an increase in activity during the fear CS. Mean and standard deviation of firing rates for baseline periods before fear CS, rewarding fluid infusion, and aversive fluid infusion, respectively: 7.4±3.3; 5.7±3.5; 5.7±2.8; (**F**) Example of a non-SD cell which had a decrease in activity during the fear CS. Mean and standard deviation of firing rates for baseline periods before fear CS, rewarding fluid infusion, and aversive fluid infusion, respectively: 0.3±0.6; 0.1±0.5; 0.2±0.4.

To determine whether the BLA encodes affective valence during learned fear, we analyzed the activity of 84 fear CS-responsive cells (cells with statistically significant responses to the fear CS and mean baseline activity >.05 Hz for all three sections of the experiment; 84/229 cells) during the fear CS, delivery of the aversive fluid, and delivery of the rewarding fluid. If the BLA encodes affective valence during learned fear, then changes in neuronal activity during the fear CS should be similar to changes in activity during the aversive fluid infusion but not to changes in activity during the rewarding fluid infusion. To test this prediction, we first computed for each cell the difference in normalized neuronal activity during the fear CS and aversive fluid infusion and averaged over all the cells – the “Aligned” condition – and compared it to what one would expect if neuronal activity during the fear CS and aversive fluid infusion were independent – the “Shuffled” condition, obtained by finding the difference in activity during the fear CS and aversive fluid infusion in arbitrary cell pairs (Fig.3A; Materials and Methods S1). Thus, if neuronal activity during the fear CS and aversive fluid infusion is similar, the difference score in the “Aligned” condition should be less than the difference score in the “Shuffled” condition, and if neuronal activity during the fear CS and aversive fluid infusion is different, the difference score in the “Aligned” condition should be greater than that in the “Shuffled” condition. Consistent with the prediction that neuronal activity during the fear CS and aversive fluid infusion is similar, the difference score in the “Aligned” condition was indeed less than the difference score in the “Shuffled” condition (Aligned, 0.47±0.03; Shuffled, 0.55±0.03; *t* test, *P*<.0001; Fig. 3B). In contrast to the comparison between the fear CS and aversive fluid infusion, there was no significant difference between the “Aligned” and “Shuffled” conditions for the comparison between the fear CS and rewarding fluid infusion (Aligned, 0.52±0.03; Shuffled, 0.56±0.03; *t* test, *P* = 0.10; Fig. 3C). Thus, neuronal activity was more similar than expected by chance for the valence-congruent comparison but not for the valence-incongruent comparison. Despite this finding, neuronal activity during the fear CS was not significantly more similar to activity during the aversive fluid infusion than activity during the rewarding fluid infusion (i.e., the direct comparison between the “Aligned” conditions was not significantly different; *t* test, *P* = .13). We reasoned that a greater similarity in neuronal activity during the two negatively valenced stimuli may have been obscured by a subpopulation of cells which encodes emotional salience/arousal independently of affective valence [9], [10], [13], [14] – cells which have qualitatively similar changes in activity during all three stimuli. To examine this possibility, we first classified fear CS-responsive cells based on the direction of their change in activity during the fear CS, aversive fluid infusion, and rewarding fluid infusion. Cells with increases or decreases in activity during all three stimuli (criterion was a minimum of 2 consecutive 1-second bins with greater than 25% absolute change in baseline firing rate) were classified as “Same Direction” (SD) cells – cells which may encode emotional salience/arousal independently of affective valence. As expected, once these putative arousal-encoding cells were removed, neuronal activity was now more similar during the fear CS and aversive fluid infusion than the fear CS and rewarding fluid infusion (Fear-Disgust, Aligned, 0.46±0.04; Fear-Reward, Aligned, 0.55±0.04; *t* test, *P* = .007; Fig. 3D). Notably, the activity of SD cells was not more similar during the fear CS and aversive fluid infusion than the fear CS and rewarding fluid infusion (SD cells only: Fear-Disgust, Aligned, 0.45±0.07; Fear-Reward, Aligned, 0.37±0.07; *t* test, *P* = .25; Fig. 3E; see Fig. 4 for population plots and individual example responses of SD and non-SD cells.). Further analysis showed that the greater similarity in activity during the two negative stimuli could not be ascribed to changes in blood pressure, heart rate, or movement during the stimuli (see Text S1). Together, these results indicate that a subpopulation of BLA neurons encodes emotional valence during learned fear.

### Timing of valence encoding

**Figure 5:**
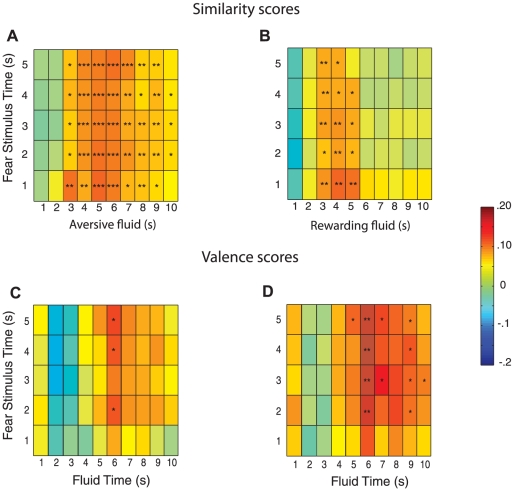
Timing of valence-specific and valence-nonspecific neuronal activity. (**A**) Valence-congruent comparison (n = 84 cells). Activity during fear CS and aversive fluid infusion is more similar than expected by chance in many time bins. (**B**) Valence-incongruent comparison (n = 84 cells). Activity during fear CS and rewarding fluid infusion is more similar than expected by chance in a smaller number of time bins towards the onset of the rewarding fluid infusion. (**C**) All fear-CS responsive cells (SD and non-SD cells; n = 84). Valence-congruent comparison of activity is more similar than valence-incongruent comparison in a small time window. (**D**) Non-SD cells (n = 62). Valence-congruent comparison of activity is more similar than valence-incongruent comparison, except towards onset of fear CS and fluid infusions. Scale bar is Z units. * *P*<.05, ** *P*<.01, *** *P*<.001.

To determine if valence encoding changed over time during the affective stimuli, we divided the activity of fear CS-responsive cells during the affective stimuli into 1-second bins and entered time during the fear CS and fluid infusions as factors in a 4-way repeated measures ANOVA, with affect type (valence congruent vs. valence incongruent) and comparison type (shuffled vs. aligned) as the other two factors. Consistent with time being an important factor for valence encoding, there was a 4-way interaction between affect type, comparison type, time during the fear CS, and time during the fluid infusion (F_83,2988_ = 1.5, *P* = .03). To further examine the effect of time on valence encoding, we calculated “Aligned” and “Shuffled” difference scores for each combination of fear CS and fluid infusion time bins. As expected, the valence-congruent comparison was more similar than expected by chance (i.e., “Aligned” scores were less than “Shuffled” scores) in many time bins (Fig. 5A). In contrast, the valence-incongruent comparison was statistically similar during a more narrow time range towards the onset of the fluid infusion (Fig. 5B). To further investigate the timing of valence encoding, we directly compared activity during the fear CS to activity during the aversive and rewarding fluid infusion (i.e., we compared the “Aligned” scores for the valence-congruent and valence-incongruent comparisons). The “Aligned” scores of the valence-congruent comparison were significantly smaller (indicating greater similarity of neuronal activity) than those of the valence-incongruent comparison in a narrow time window when all fear-CS responsive cells were included in the analysis (Fig. 5C) but in a much wider time window when SD cells were excluded (Fig. 5D). Interestingly, even with SD cells excluded, the “Aligned” scores of the valence-congruent comparison were not statistically different from those of the valence-incongruent comparison at the onset of the fear CS and fluid infusions (Fig. 5D). Together, these results indicate that valence-nonspecific activity–activity that may be important for emotional salience/arousal–is greater at the onset of affective stimuli, while valence-specific activity is greater during later time windows.

### Characterization of fear CS-responsive cells

In agreement with our previous study [14], we also found that fear CS-responsive cells with increases in firing rate during the fear CS had, on average, faster baseline firing rates than cells with decreases in firing rate during the fear CS (t test, *P* = .007), as well as shorter spike durations (t test, *P* = .00003)(Fig. S3). This was true for both SD cells and non-SD cells (all P<.05). Because interneurons have, on average, faster baseline firing rates and shorter duration action potentials than projection neurons [30], [31], these findings suggest that cells with increases in activity during the fear CS are more likely to be interneurons than cells with decreases in activity. Future studies that identify the cell type of the recorded neuron within these behavioral procedures are needed to further explore this possibility.

## Discussion

In this study we tested and confirmed a critical prediction of the hypothesis that the BLA encodes emotional valence: that neuronal activity during two different affects of the same valence - fear and disgust - is more similar than neuronal activity during two affects of different valence – fear and pleasure. We further show that valence-specific activity in the BLA is restricted to a subpopulation of neurons, as well as a limited time window after the onset of the affective stimuli.

Given that emotional salience/arousal (also called affective intensity) and emotional valence are the two most commonly described dimensions of emotion [32], our results clearly support a dimensional approach to understanding the contribution of the BLA to emotional processing, as do several previous studies [8]–[14]. Nevertheless, our results do not rule out the possibility that the BLA also encodes discrete affects, such as fear and disgust, differently, since the patterns of neuronal responses during the fear CS and aversive fluid infusion were clearly not identical (Fig. 3B). It remains to be determined whether these differences in neuronal activity reflect differential encoding of two different affects, the different sensory properties of each of the stimuli, or different autonomic and/or motor responses induced by the stimuli.

Our results build on those of prior studies which showed that the responses of many amygdala neurons to negatively and positively valenced conditioned stimuli change if the valence of the stimuli is changed via reversal of their associated outcomes [8], [12] or conditioning [19], [21], [22], as well as studies that found cells with opposite responses to a negatively valenced stimulus and positively valenced stimulus [9], [14], [33]. Our experiments extend these findings to neuronal activity during learned fear and rule out alternative explanations to valence encoding – that the differences in neuronal activity were only due to differences in the specific affect induced by the stimuli, the sensory properties of the associated outcomes, or changes in arousal. Our results also build on a previous recording study in monkeys which reported that some amygdala neurons only responded to the presentation of negatively valenced stimuli [10]. The nature of the affective responses during these stimuli was not characterized, however, so it remained unclear whether amygdala neurons respond similarly during two different negatively valenced affects.

Notably, neuronal activity of SD cells was not more similar during the two negative stimuli than during the fear CS and the rewarding fluid. This suggests that valence encoding does not occur throughout the entire BLA but instead is restricted to a subpopulation of cells, consistent with previous results [8]–[10], [12], [13]. We also found that valence-nonspecific activity was greatest at the beginning of the affective stimuli, while valence-specific activity was larger during later time windows. The function of valence-nonspecific activity may be to prepare the animal to respond quickly to biologically important stimuli [14], [34], while valence-specific activity may be important for online calculation and/or storage of stimulus value used to guide decision-making [8], [35]–[40]. A related temporal dissociation between salience and valence was recently found in midbrain presumed dopamine neurons [41]. In this study, dopamine neurons first responded in a valence-nonspecific way to a visual fixation stimulus which signaled trial onset, but then responded in a valence-specific way to a second stimulus that signaled trial outcome. Thus, initial valence-nonspecific activity followed by increasing valence-specific activity may be a general principle of affective stimulus processing that occurs in several brain regions, although we cannot rule out the possibility that the particular sensory qualities of the stimuli used in this study affected our ability to identify salience and valence encoding.

We have shown that the activity of a subpopulation of BLA neurons is consistent with emotional valence encoding during learned fear, and that this valence specific activity is time-dependent. Furthermore, because oral rejection responses are considered to be the foundation of other forms of disgust [42]–[44], we believe that our results during the aversive fluid infusion are relevant to the encoding of multiple forms of disgust in humans. Thus, our results suggest that fear and disgust are encoded similarly in the amygdala, in part because of their shared negative emotional valence.

## Materials and Methods

### Subjects

Seven male Long-Evans rats (325–400 g at the time of first surgery) were housed individually under a 12 hour light/dark cycle and given access to food *ad libitum* throughout the experiment. Water access was restricted for ∼21 hours before each behavioral session to enhance positive hedonic taste reactivity to the rewarding fluid during the test sessions and to increase consumption during the taste aversion conditioning sessions.

### Surgery

Rats were anesthetized with isoflurane and implanted abdominally with a telemetric transmitter for measuring blood pressure and movement (Data Sciences International; St. Paul, MN). Six of the rats were then implanted with 5-electrode driveable arrays unilaterally into the amygdala and three intraoral cannulae after 10–14 days of recovery from the first surgery. The remaining rat was implanted bilaterally with fixed 8-electrode arrays instead of the driveable array. For the intraoral cannula and electrode surgeries, a mixture of ketamine (100 mg/kg) and xylazine (10 mg/kg) was used initially for anesthesia, followed by isoflurane as needed. Stereotaxic coordinates were A-P: −3.0 mm posterior; M-L: 4.85–5.0 mm; D-V: 6.7–7.0 mm for driveable arrays and 7.75 mm for fixed arrays (ventral from the surface of the brain) relative to bregma. Training began after 10–15 days of recovery in the home cage. This study was carried out in strict accordance with the recommendations in the Guide for the Care and Use of Laboratory Animals of the National Institutes of Health. The protocol was approved by the Ernest Gallo Clinic and Research Center Institutional Animal Care and Use Committee (Protocol Number: 07.04.147).

### Analysis of Neural Data

Neurons were considered to be fear CS-responsive if their spiking activity during the 5 second CS interval was different than their activity during the 10 seconds preceding CS onset (Wilcoxon signed-rank test, α = .05). Neurons with extremely low baseline firing rates (less than .05 Hz) during any of the three sections of the experiment (fear conditioning section, rewarding taste reactivity section, or aversive taste reactivity section) were excluded from all analyses (10 out of 94 fear CS-responsive cells).

For more information on materials and methods, please see Materials and Methods S1.

## Supporting Information

Figure S1 **Speed of intraoral infusions.** The infusion rates of three lines were calculated by averaging the rates of ten infusions per line. The smoothed averages are shown here. The first ten seconds of the infusions were used for all neuronal analyses since this block of time included the onset and peak rates of the infusions.
(EPS)

Figure S2 **Movement during fear CS during conditioning sessions.** There was an increase in movement, on average, during the fear CS, reflecting the escape/fleeing-like reactions that occurred during the conditioning sessions (n = 39 sessions).
(EPS)

Figure S3 **Baseline firing rates and spike durations of fear CS-responsive cells.** Cells with increases in activity during the fear CS had, on average, higher baseline firing rates and smaller spike widths.
(EPS)

Text S1 **Changes in blood pressure, heart rate, and movement do not account for valence encoding.**
(DOC)

Materials and Methods S1 **Supplementary explanation of experimental procedures.**
(DOC)
