# Supplementary material for: Similar Neural Activity during Fear and Disgust in the Rat Basolateral Amygdala
Source: PLoS One. 2011 Dec 14;6(12):e27797. doi: 10.1371/journal.pone.0027797 (PMC3237420; doi:10.1371/journal.pone.0027797)
Supplement: Text S1 — Changes in blood pressure, heart rate, and movement do not account for valence encoding. (DOC) [file pone.0027797.s004.doc]

**Text S1:**

The greater similarity in changes in neuronal activity during the fear CS and aversive fluid infusion compared to the fear CS and rewarding fluid infusion is unlikely to be due to changes in blood pressure during the three stimuli, since changes in blood pressure were more similar during the fear CS and rewarding fluid infusion than the fear CS and the aversive fluid infusion (though not significantly so; see Fig. 2A and main text).

We also investigated changes in heart rate and movement to see if these factors could have accounted for the greater similarity in changes in neuronal activity during the fear CS and aversive fluid infusion compared to the fear CS and rewarding fluid infusion. Consistent with this possibility, changes in movement and heart rate were more similar during the fear CS and aversive fluid infusion than during the fear CS and rewarding fluid infusion (∆ movement counts: fear CS, .32 ± .04, aversive fluid, .03 ± .01, rewarding fluid, -.77 ± .14; ∆ heart rate, beats/s: fear CS, .39 ± .04, aversive fluid, .006 ± .014, rewarding fluid, -.052 ± .017). To further investigate these issues, we categorized fear CS trials depending on whether there was an increase in movement or heart rate during the trial, resulting in two sets of trial categories – increased movement vs. decrease/no change movement trials and increased heart rate vs. decrease heart rate trials. We then reanalyzed the activity of fear CS-responsive cells (excluding SD cells) to see if the greater similarity in neuronal activity during the two negatively valenced stimuli (compared to during the fear CS and rewarding fluid infusion) was enhanced during increased movement or increased heart rate trials, relative to decreased/no change movement and decreased heart rate trials, respectively. This was not the case for the movement comparison (valence-encoding score: movement increase, .066 ± .0409, movement decrease/no change, .085 ± .0416) nor the heart rate comparison (valence-encoding score: increased heart rate, .12 ± .052, decreased heart rate, .11 ± .052; t test, P > .38). Thus, the valence-congruent neuronal activity cannot be ascribed to changes in movement or heart rate.
