# Supplementary material for: Similar Neural Activity during Fear and Disgust in the Rat Basolateral Amygdala
Source: PLoS One. 2011 Dec 14;6(12):e27797. doi: 10.1371/journal.pone.0027797 (PMC3237420; doi:10.1371/journal.pone.0027797)
Supplement: Materials and Methods S1 — Supplementary explanation of experimental procedures. (DOC) [file pone.0027797.s005.doc]

**Materials and Methods S1:**

**Surgery.** For intraoral cannula implantation, we used methods given to us by Dr. Kyle S. Smith, formerly of Dr. Kent Berridge’s lab at the University of Michigan. Briefly, heat-flared, antibiotic-coated PE100 tubing was threaded through the cheek lateral to the first molar and out the top of the head with a 23 gauge needle. A small, flexible rubber washer (Metro Industries) prevented the heat-flared end (~ 3 mm long) from entering the cheek. A 1-2 cm long piece of 23 gauge steel tubing was inserted and glued into the PE100 tubing at the top of the head. The steel tubing was then glued to a small plastic cone that was cemented to the rat’s skull to protect the electrode assembly.

**Electrode Assemblies**. The driveable electrode assemblies consisted of five 50 μm Teflon-coated stainless steel wires (California Fine Wire; Grover Beach, CA) of equal lengths bundled into a 25 gauge cannula and attached to a driveable connector. The wires were advanced ventrally by turning a screw located in the connector. Before implantation, the wires were arranged into a single row ~ 1 mm wide and placed in brain so they extended primarily in the anterior-posterior direction. The fixed electrode arrays were bundles of eight 50 μm Teflon-coated stainless steel wires (NB Labs). The fixed electrode arrays were cemented to the skull during surgery. Before each recording session, the wire with the least neuronal activity was used as an internal reference to minimize background activity on the remaining wires. A 200 μm Teflon-coated stainless steel wire (A-M Systems; Carlsborg, WA) stripped ~1 mm from the tip was placed it in the posterior cortex for use as an animal ground.

**Behavioral Equipment.**  Fear conditioning and taste reactivity conditioning and testing occurred in the same sound attenuating chamber for each rat. However, fear conditioning took place in a custom 31 x 33 x 29 cm (length x width x height) Plexiglas square with a grid floor, whereas taste reactivity took place in a 25 cm diameter, 25 cm tall Plexiglas tube that was placed inside the Plexiglas square after the floor and ceiling were removed. A high-frequency “tweeter” speaker (ENV-224BM) and houselight (28V, 100 mA) were located 20 cm from the floor. The footshock was delivered through the grid floor by a constant current aversive stimulator (ENV-414S). All of the above (not including the Plexiglas tube) were obtained from Med Associates, Inc. (St. Albans, VT). Movement and blood pressure data were sent to the neural data acquisition system from a receiver (Data Sciences International) located underneath the chamber floor during fear conditioning or to the side of the Plexiglas tube during taste reactivity. A video camera pointed through a small hole cut into the sound-attenuating shell was used to monitor the animal’s behavior during fear conditioning.

To monitor taste reactivity responses, a circular mirror was angled below the floor of the Plexiglas tube so that the rat’s mouth was visible to a camera mounted on a miniature tripod in the sound attenuating chamber. The floor underneath the Plexiglas tube and the ceiling were also made of Plexiglas. A hole was cut in the center of the ceiling for the electrophysiology cable and taste reactivity tubing.

**Behavioral Procedures and Analysis.** Rats were first given a tone habituation session to measure baseline changes in blood pressure during the tone that would later be used during fear conditioning. This session consisted of 20 presentations of the tone (5 seconds duration, 3 kHz, 85 dB, 10 ms rise/fall, 90 second mean ITI) without footshock. Next, rats were given 6 taste reactivity sessions to establish a discriminative conditioned taste aversion to the aversive fluid. Each session consisted of 20 rewarding or aversive fluid deliveries (~ 75 microliters, 90 second mean ITI, 0.9% w/v NaCl plus 1% v/v almond flavoring or 10% w/v sucrose plus 1% v/v banana flavoring) followed by a 2-3 minute continuous infusion at ~ 0.5 mL/min. The first, third, and fifth sessions consisted of rewarding fluid deliveries (sucrose solution for 4 rats and salt solution for 3 rats) and the second, fourth, and sixth sessions consisted of aversive fluid deliveries (salt solution for 4 rats and sucrose solution for 3 rats). The aversive fluid conditioning sessions were always followed ~ 10 minutes later by an intraperitoneal injection of 20 mL/kg 0.15 M LiCl solution. The rewarding fluid conditioning sessions were always followed ~ 10 minutes later by an intraperitoneal injection of 20 mL/kg 0.9% NaCl. Six to seven daily testing sessions followed the taste reactivity conditioning sessions. Each testing session consisted of three parts – a fear conditioning section, a rewarding taste reactivity section, and an aversive taste reactivity section. Three rats had testing sessions that began with the rewarding taste reactivity section, followed by the aversive taste reactivity section and then the fear conditioning section, and four rats had testing sessions that began with the fear conditioning section, followed by the rewarding taste reactivity section and then the aversive taste reactivity section. Each fear conditioning section was identical to the tone habituation session, except that the termination of each of the 20 tones was always followed by mild footshock (0.5 s, 0.4 mA). Each rewarding taste reactivity section consisted of 20 presentations of the rewarding fluid (~ 75 microliters, 90 second mean ITI) interspersed with 20 water rinses (~ 75 microliters, 90 second mean ITI). Each aversive taste reactivity section consisted of 20 presentations of the aversive fluid (~ 75 microliters, 90 second mean ITI) interspersed with 20 water rinses (~ 75 microliters, 90 second mean ITI). Between the fear conditioning and taste reactivity sections, rats were removed from the chambers and kept in their home cages for ~ 20 minutes while the chambers were prepared for the next section of the experiment. The recording cables and headstages were not detached from the rat during this time or at any time during the experiment. After each testing session, rats were given ~90 minutes access to water in their home cages. To prevent extinction of the conditioned taste aversion, after each block of 6-7 testing sessions, rats were given 2 additional taste reactivity conditioning sessions, a rewarding conditioning session (paired with a saline injection) followed the next day by an aversive taste reactivity conditioning session (paired with a lithium chloride injection). Each rat received 2-3 blocks of testing sessions.

The data shown here were collected from 45 testing sessions from 7 rats (median: 7 sessions/rat; range: 1-9 sessions/rat). All sessions in which the electrodes were calculated to be in the BLA were included, with the exception of one rat that was implanted with fixed electrode arrays. For this rat, only the session with the most isolated single units was included in the analyses. None of the testing sessions that were included in the analyses was the first or second testing session, so all included data were collected after at least 40 fear conditioning trials.

At the end of the experiment, rats were tested for freezing during the fear CS in the same chamber but with novel contextual cues (visual, olfactory, and tactile). Three minutes after placing the rat in the chamber, the Shock CS+ was presented once for 1 minute and freezing was scored offline via videotape. Because most freezing occurred during the first ten seconds of the fear CS during the test, we only compared the amount of freezing during the ten seconds prior to the start of the fear CS and the ten seconds after the start of the fear CS. Rats were considered to be freezing if they were completely immobile (except for breathing) for one full second.

Taste reactivity responses were scored offline by observers (R.D. and V.P.) that were blind to the identity of the fluid. All analyzed recording sessions (n = 45) were scored during the 10 seconds before and 20 seconds after the start of the infusions. For each 5 second block, the presence or absence of each taste reactivity response was recorded. Because 3 types of positive hedonic responses (tongue protrusions, paw licking, and lateral tongue protrusions) and 5 types of aversive responses (gaping, head shaking, forelimb flailing, paw treading, and chin rubbing) were monitored, the maximum score for each time bin was 3 and 5, respectively, for each trial and 60 and 100, respectively for each session.

**Blood Pressure and Movement.** Mean arterial pressure was used in all blood pressure analyses. Long-lasting artifactual drops or increases in the blood pressure signal were removed and treated as missing values in the dataset. If only one arterial pressure value was corrupted, then the value was replaced with the average of the immediately surrounding values. Because the catheter from one rat malfunctioned during the experiment, blood pressure and movement data from these sessions (6 total sessions) were excluded from the analyses. Changes in blood pressure were calculated by subtracting the mean blood pressure during the ten seconds preceding the onset of the stimulus from the interval of interest.

Movement was measured in “movement counts” generated by the blood pressure transmitter. Any movement of the rat which resulted in sufficient translocation of the transmitter produced a movement count. Since it was an arbitrary value, we used it as a relative measure. Fear CS trials with increases, decreases, or no change in movement were determined by comparing the number of movement counts during the CS to the number of movement counts during the ten seconds preceding the CS.

**Electrophysiological Methods**. Neuronal activity was recorded with commercial hardware and software, including headstage amplifiers and programmable amplifiers, filters (0.4 and 5 KHz), and multichannel spike-sorting software (Plexon Inc.; Dallas, TX). Discrimination of individual units was performed offline using principal component analysis of waveform shape. Single cells were identified by constancy of waveform shape, cross-correlograms, and interspike intervals[1]. Action potential durations were measured from the first trough to the first peak. Cells with peak-then-trough spike shapes (5 of 84 cells) were excluded from the spike duration analysis because of difficulty measuring the time at peak.

At the beginning of each recording session, the wires were screened for identifiable single-cell activity. If none was found, the wires were advanced 80 μm and the animal was put back into its home cage. If identifiable single-cell activity was present on any of the wires, a recording session was conducted. After the recording session, electrodes were advanced 80-120 μm to acquire activity from new cells for the following day. Recording ceased for each rat when the estimated position of the electrodes was ventral to the BLA.

**Analysis of Neural Data.** “Same direction” cells were fear CS-responsive cells which had at least 25% increases in activity during the fear CS, the rewarding fluid infusion, and the aversive fluid infusion for at least two consecutive 1-second bins or at least 25% decreases in activity during fear CS, the rewarding fluid infusion, and the aversive fluid infusion for at least two consecutive 1-second bins.

Z scores were calculated using the formula [(activity during time bin) – (mean activity of cell)]/[standard deviation of activity of cell], where the mean activity was computed during the 60 seconds preceding the ten second baseline period and the standard deviation was computed across the entire corresponding section of the experiment (fear conditioning section for fear CS, taste reactivity sections for rewarding and aversive fluid infusions). Note that for all of the analyses described here, the particular interval used to compute mean activity is irrelevant since neuronal activity changes were computed by subtracting the mean neuronal activity during the ten seconds preceding the onset of the CS from the interval of interest. Changes in Z scores rather than absolute Z scores were used to be consistent with the blood pressure and taste reactivity analyses, to equate baselines for different trial types, and to minimize the effects of spike shape drift during the experiment.

To determine whether neuronal activity was more similar than expected by chance during the fear CS and the aversive/rewarding fluid infusions, we first calculated randomized difference scores between ∆Z scores during the fear CS and fluid infusions. The randomized scores were computed by first shuffling the ∆Z scores for one of the two stimuli 1000 times and then taking the difference in ∆Z scores for each “cell pair” (one ∆Z score for one cell during one stimulus subtracted from one ∆Z score for a different cell during the other stimulus) for each shuffle and averaging over all 1000 shuffles for each cell. This chance value was then compared to the actual value, computed in the same way except without the shuffling, to determine if neuronal activity was more similar than expected by chance during the fear CS and aversive/rewarding fluid infusion.

**Histology.** Rats were deeply anesthetized with sodium pentobarbital and the final location of the wires was marked by passing current (19 μA; 10-15 s). The brains were cut into 50 μM sections and stained with neutral red. To estimate the location of cells recorded on a given day, we followed the electrode tracks from the tips of the electrodes dorsally, using the known travel distance after each session (margin of error in each direction is 100-300 μM). Using the atlas of Paxinos and Watson (1998) as a reference, 229 cells were determined to be in the BLA (basal and lateral nuclei).

Supporting Information Reference:

1. Janak PH (2002) Multichannel neural ensemble recording during alcohol self-administration*. Methods in Alcohol-Related Neuroscience Resear*ch, eds Liu Y & Lovinger DM (CRC Press, Boca Raton), pp 241-258.
